# Complete Genome Sequence of *Enterococcus faecium* ATCC 700221

**DOI:** 10.1128/genomeA.00386-16

**Published:** 2016-05-19

**Authors:** Peter T. McKenney, Lilan Ling, Guilin Wang, Shrikant Mane, Eric G. Pamer

**Affiliations:** a Immunology Program, Infectious Diseases Service, Department of Medicine, Lucille Castori Center for Microbes, Inflammation, and Cancer, Sloan Kettering Institute, Memorial Sloan Kettering Cancer Center, New York City, New York, USA; b Yale Center for Genome Analysis, W. M. Keck Biotechnology Resource Laboratory, Yale University School of Medicine, Yale University, New Haven, Connecticut, USA

## Abstract

We report the complete genome sequence of a vancomycin-resistant isolate of *Enterococcus faecium* derived from human feces. The genome comprises one chromosome of 2.9 Mb and three plasmids. The strain harbors a plasmid-borne *vanA-*type vancomycin resistance locus and is a member of multilocus sequencing type (MLST) cluster ST-17.

## GENOME ANNOUNCEMENT

*Enterococcus faecium* ATCC 700221 is a *vanA*-positive fecal vancomycin-resistant enterococcus (VRE) isolate with high levels of resistance to several antibiotics, and it has been used in animal models of VRE intestinal colonization (1, 2). We produced a complete genome sequence of the strain in order to facilitate studies of genome dynamics and mutation accumulation during infection.

Colonies were grown overnight to exhaustion in brain heart infusion (BHI) liquid medium. DNA was extracted using phenol-chloroform extraction with bead beating and purified with AMPure XP beads. Libraries of 10 Kb were prepared by standard PacBio protocols (P/N 100-266-000-05) with BluePippin size selection. The samples were loaded with MagPure bead capture, sequencing was performed with P6-C4 chemistry, and data were captured for 4 h. For Illumina sequencing, purified DNA was sheared using a Covaris ultrasonicator and prepared for sequencing with a Kapa library preparation kit with Illumina TruSeq adapters to create 300 × 300-bp nonoverlapping paired-end reads (3).

PacBio reads from 2 single-molecule real-time (SMRT) cells were assembled in SMRT Analysis using HGAP_3 with default settings (4), yielding 4 contigs. They include a chromosome of 2.9 Mb and candidate plasmids of 189 kb, 64 kb, and 39 kb, respectively. The consensus was realigned to the PacBio reads using RS_resequencing_1 in SMRT Analysis, iteratively, for three rounds of self-correction by the Quiver algorithm, resulting in a consensus accuracy that was >99.999% for all contigs. PacBio data are known to be susceptible to single-base deletions in homopolymer runs during the calculation of circular consensus reads, and alignment of the Illumina reads to the PacBio consensus revealed 561 such deletions. We used Pilon to correct the PacBio consensus with the Illumina reads (5), which eliminated >90% of these deletions.

The genome was annotated using the NCBI Prokaryotic Genome Annotation Pipeline (6). ATCC 700221 contains 2,725 predicted genes and belongs to multilocus sequencing type (MLST) cluster ST-17 (7, 8).

Plasmid-1 is 99% identical to pDO3 from *E. faecium* DO over 61% of its length (7) and contains a predicted *hyl_Efm_* gene and *pilA–fms21-22* pilin gene cluster, which are thought to be virulence factors in mouse models (9–11). Plasmid-2 is 99% identical to the conjugative plasmid pMG1 over its entire length (12), and plasmid-3 is 99% identical to the Tn*1546-*containing VanA resistance plasmid pIS177 over 95% of its length (13). The VanA locus contains a predicted IS*1251* family insertion sequence between *vanRS* and *vanHAXYZ* (14).

PHAST predicts 6 complete prophages in ATCC 700221 (15). We note the presence of a large predicted prophage island between 1,624,385 bp and 1,729,248 bp. In this region, the average mapping quality values of PacBio reads aligned to the consensus is low, while the overall coverage is about 0.5 times greater (~450×) than that of the rest of the genome (~300×). This might indicate misassembly of a repetitive region. However, it is completely spanned by long reads with high mapping quality values, suggesting that the assembly may nonetheless be accurate in this region.

### Nucleotide sequence accession numbers. 

The assembled sequences were submitted to GenBank under the accession numbers CP014449, CP014450, CP014451, and CP014452. The versions described in this announcement are the first versions.
